# Current patterns of primary care provider practices for the treatment of post-traumatic headache in active duty military settings

**DOI:** 10.1371/journal.pone.0236762

**Published:** 2020-07-24

**Authors:** Rosemay A. Remigio-Baker, Seth Kiser, Hamid Ferdosi, Emma Gregory, Scot Engel, Sean Sebesta, Daniel Beauchamp, Saafan Malik, Ann I. Scher, Sidney R. Hinds

**Affiliations:** 1 Defense and Veterans Brain Injury Center, Silver Spring, MD, United States of America; 2 Henry Jackson Foundation for the Advancement of Military Medicine, Bethesda, MD, United States of America; 3 Naval Hospital Camp Pendleton, Camp Pendleton, CA, United States of America; 4 General Dynamics Information Technology, Falls Church, VA, United States of America; 5 Fort Hood, Intrepid Spirit Center, Ft Hood, TX, United States of America; 6 Fort Bliss, Intrepid Spirit Center, Ft Bliss, TX, United States of America; 7 Preventive Medicine & Biostatistics, Uniformed Services University, Bethesda, MD, United States of America; 8 Neurology, Uniformed Services University, Bethesda, MD, United States of America; University of Florida, UNITED STATES

## Abstract

**Objective:**

To provide a preliminary assessment of the current clinical practice for the treatment of post-traumatic headache following concussion in military primary health care settings.

**Background:**

Headache is one of the most common symptoms post-concussion; however, little is known of the current clinical practices of primary care providers (on the treatment of post-traumatic headache), particularly in military settings.

**Methods:**

Study participants were primary care providers (n = 65) who treated active duty Service members suffering from post-traumatic headache at two military installations. Qualitative data gathered via semi-structured interviews were used to describe provider practices and experience in treating patients with post-traumatic headache.

**Results:**

Some patterns of care across primary care providers treating post-traumatic headache were consistent with the Department of Defense-recommended clinical recommendation (e.g., recommendation of both pharmacological and non-pharmacological treatment [89.4%]; engaging in follow-up care [100%]). Differences existed in timing of follow-up from initial visit [16.9% reporting within 24 hours; 21.5% reporting within 48–72 hours; and 26.2% reporting more than 1 week], the factors contributing to the type of care given (e.g., symptomatology [33.0%], injury characteristic [24.2%], patient characteristic [13.2%]) and the need for referral to higher level of care (e.g., symptomatology [44.6%], treatment failure [25.0%]). These variations may be indicative of individualized treatment which would be compliant with best clinical practice.

**Conclusion:**

The results of this study demonstrate the current clinical practice in military primary care settings for the treatment of post-traumatic headache which can potentially inform and improve implementation of provider training and education.

## Introduction

Post-traumatic headache (PTH) is a common sequela of mild traumatic brain injury (mTBI), also known as a concussion [1–5]. A prospective study previously reported a cumulative incidence of 91% for new onset or worsening headaches over one year post-concussion, with persistent headache reported for over a third of the study participants [5]. The majority of PTH improve within 6 to 12 months post-injury; however, 18–33% may continue well beyond a year [3], and nearly 25% persist for more than a year [6]. Although PTH can vary in type, the most common of which are phenotypically migraine-like followed by tension-type or other headaches such as cervicogenic headaches [3, 5, 7, 8], PTH in general has debilitating and disruptive effects on normal daily functioning [9–11].

The military population may be especially vulnerable to the impact of traumatic brain injury given the demands of their physical fitness requirements, military training and the rigors of armed combat. With over 340,000 Service members (SMs) having been diagnosed with mTBI since 2000 [12], TBI has a tremendous impact on deployability and force readiness. In a study of veterans from Operation Enduring Freedom/Operation Iraqi Freedom, a reported 74% had PTH occur within 30 days of concussion [13]. While full recovery for mTBI among SMs are usually within 3 months, a substantial number (47%) has been shown to still exhibit post-concussive symptoms, which includes PTH (15%), beyond this period [14]. Given the prevalence of PTH among those who have suffered mTBI, proper management of such a highly prevalent event becomes of utmost importance in facilitating and expediting return to duty among SMs to the extent possible [15, 16].

Overall care for PTH is similar to treatment for primary headache with special consideration of particular red flags associated with mTBI (e.g., Glasgow Coma Scale Score < 15, loss of consciousness > 5 minutes, repeated vomiting, thunderclap headache [sudden onset], sudden neurological deficit, presence of systemic symptoms) [17]. It requires understanding of headache history and diagnosis of specific headache type(s) to ensure appropriate management, as well as the use of both pharmacological and non-pharmacological treatment approaches. When seeking medical care, primary care providers (PCPs) are often the first point of contact for those who have sustained a mTBI. They are tasked not only with managing all of the medical aspects resulting from mTBI, but also with understanding and managing other pre-existing and co-morbid medical conditions and within short periods of time per clinical visit. Perhaps due to a variety of reasons (e.g., lack of specific training on PTH; insufficient time with patients), PCPs may be quick or late to refer patients to higher levels of care, oversimplify diagnosis (e.g., PTH vs. migraine PTH), not obtain a complete headache history, focus on pharmacological or non-pharmacological approaches rather than consider a combined treatment, or provide only limited patient education that is critical to PTH treatment and management. Clear standard of care protocols and dissemination of those protocols are required to ensure that providers are familiar with and able to implement the most current recommendations to treat PTH. Even when factoring in individual patient differences, a standard of care will serve as a clear foundation from which to guide PCPs treating PTH in a military setting.

In the US military, specific guidance on PTH management indeed exists for PCPs. Based on expert opinion, the Defense and Veterans Brain Injury Center-led Department of Defense (DoD) Clinical Recommendation (CR) on PTH provides clinical guidance for PCPs in both deployed and non-deployed settings for managing PTH following mTBI: evaluating, diagnosing and treating PTH using both pharmacological and non-pharmacological approach [18–20]. These clinical recommendations were created at the request of providers in the armed services to address a gap in knowledge regarding PTH management; however, it is uncertain to what extent clinicians are aware of these guidelines.

Despite the public availability of the DoD recommendations, training is critical to support dissemination and implementation. Moreover, ensuring providers can implement this knowledge into practice is complex in military medical settings. Uniformed military medical providers move regularly, oftentimes fairly abruptly, which may suspend or even terminate training/education. As is typical in the military setting, assignments change in response to changing operational requirements. These military requirements make it challenging to guarantee that all providers who may treat PTH receive continued and sufficient training. These obstacles emphasize the need to better understand the general knowledge of PCPs in the military, the care provided, and the experience of PCPs with identifying and managing PTH and mTBI as a means to better tailor medical training specific to these health issues.

The aims of this study were to describe: 1) the current clinical practices of PCPs for managing PTH (e.g., pharmacological and non-pharmacological treatment intervention recommendations, provision of patient education and pathways of care such as referrals); 2) provider experience in treating PTH patients; and 3) the comfort level in treating these patients. As a secondary aim, this study examined the providers’ perceived challenges for treating PTH from the perspective of the providers and the larger clinical system. The results of this study provided a preliminary account of the current clinical practices of PCPs in the management of PTH, whether those aligned with clinical recommendations (e.g., use of both pharmacological and non-pharmacological treatment approach, following-up with patients), and areas for improvement for future care.

## Materials and methods

### Study participants

The study’s provider participants were identified and recruited from several clinics where concussed SMs seek care for their clinical management at two locations: the Carl R. Darnall Army Medical Center at Fort Hood, and the William Beaumont Army Medical Center at Fort Bliss. Participants for this study were all medical staff members, including physicians, physician assistants, and nurse practitioners who provided care for concussed SMs and who worked at one of these two U.S. Army military treatment facilities. Providers learned about the study through face-to-face encounters with a study investigator, regular clinic meetings where study announcements were made informing or reminding attendees about local military treatment facilities research opportunities and research staff points of contact, and/or approved research flyers posted in clinics.

### Study design and protocol

This investigation included qualitative data derived from semi-structured interviews with PCPs treating PTH in active duty SMs from January 2018 to August 2018. This qualitative research followed the Consolidated Criteria for Reporting Qualitative Research guidelines [21]. During the consent process, the research staff (consisting of research assistants, associates and coordinators with Bachelors and Masters degrees) described the study protocol, specifying that participation is voluntary, not mandatory, and not command-directed. Shortly after completing written informed consent, the research staff conducted the first provider interview, as outlined in the study protocol. The study was reviewed and approved by the Brooke Army Medical Center Human Research Protections Office, and the protocols used in the study complied with all applicable regulations through implementation of the Brooke Army Medical Center Human Research Protection Program and administration of the Regional Health Command-Central Institutional Review Board.

#### Interview structure, content and implementation

Data were obtained via a semi-structured interview conducted in-person by research staff in a dedicated space (e.g., exam room, clinician office) and lasting, on average, 20 minutes. Prior to the study and to support standardization across interviewers and sites, all interviewers were specifically trained on a detailed study operating manual outlining details of the questionnaires, interview techniques, and ways to respond to interviewee comments or questions. Additionally, they underwent multiple practice sessions to ensure proper administration to participants during the conduct of the study. All interview data were collected in person and in individual one-on-one sessions. To avoid introducing interviewer bias, we used standardized questionnaires consisting of both open- and closed-ended questions, as well as trained the interviewer to adhere to the question an answer format strictly. No relationship was established with participants to prevent knowledge of the study. Interviews were recorded using a Phillips DVT 6000 recorder. After completion of the interviews, the research staff transcribed the recording, and another member of the staff reviewed and verified the transcription. The same questions were asked of all participants, with follow-up questions permitted as needed to allow for elaboration of participant responses. Based on the answers provided to the open-ended questions of the semi-structured interview, all provided responses were identified, tabulated and categorized as appropriate based on content analysis [21]. Multiple responses to open-ended questions regarding current clinical care, with the exception of recommended follow-up, were possible from each provider.

**Table in S1 Table** organizes questions included in the semi-structured provider interview into 3 key categories: 1) background or experience (e.g., number of concussion patients seen; years practicing); 2) current care provided to PTH patients (e.g., recommended pharmacological and non-pharmacological treatments; factors in determining when to refer a patient to a rehabilitation provider); and 3) perception of patient change as a result of the care, and perception of patient compliance with provider recommendations.

#### Data dictionary and coding variables

A data dictionary was created to code transcribed provider responses. A deductive approach was used to develop the data dictionary where the transcribed responses were assigned to pre-determined codes, which were theoretically- or empirically-based. This iterative process involved the investigator team, where two team members coded each transcript. Raters were required to reach consensus through discussion for any identified discrepancies. Any discrepancy amongst reviewers was resolved through discussion among the research group until consensus was reached. Coded responses were further refined by combining similar response categories into newly coded variables (e.g., patient-related factors) and eliminating responses that were endorsed by fewer than 5% of respondents. Newly-coded variables were created based on consensus from separate members of the research team with expertise in psychology and epidemiology.

#### Analyses

Descriptive statistics were presented as means (standard deviations [SD]) or medians (interquartile range [IQR = 25^th^, 75^th^]) for continuous variables depending on normality of data, and as frequencies (percentages) for categorical variables. Due to the exploratory and descriptive nature of this study, significance testing was not conducted. All analyses were completed using Stata statistical software, release 15 (StataCorp, 2017, College Station, TX).

## Results

### Provider background

**Table 1** summarizes the characteristics of provider participants including their experience treating concussion patients with headaches. Of the 65 PCPs in the study, 84.6% were uniformed military providers and 15.4% were civilian. There were 61.5% (n = 40) physician’s assistants, 29.2% (n = 19) physicians and 9.2% (n = 6) nurse practitioners. The median number of years caring for patients with PTH was reported to be 2.5 years (IQR = 1.5, 6), which was similar to the median of years practicing medicine (3 years, IQR = 2, 6). The providers in the study had a median of 1 concussion patients treated per month (IQR = 0.5, 3.3), with a median of 6 patients (IQR = 4, 14) treated with headache, on average.

**Table 1:** Provider characteristics and experience treating concussion and concussion-related headache among Service members (N = 65).

Variable	Frequency (%) or Medians (IQR)	Range
**Site, n (%)**		
Fort Hood	34 (52.3)	---
Fort Bliss	31 (47.7)	---
**Status, n (%)**		
Military	55 (84.6)	---
Civilian	10 (15.4)	---
**Professional Role, n (%)**		
Physician’s Assistant	40 (61.5)	---
Physician	19 (29.2)	---
Nurse Practitioner	6 (9.2)	---
**Number of Years Practicing, median (IQR)**	3 (2, 6)	0.5–30
**Number of Years Treating Patients with Concussion-related Headache, median (IQR)**	2.5 (1.5, 6)	0–27
**Number of Headache Patients Treated per month, median (IQR)**	6 (3, 14)	0–60
**Number of Concussion Patients Treated per month, median (IQR)**	2 (0.5, 2.8)	0–32
**Number of Patients with acute concussion treated overall, median (IQR)**	12.5 (5, 30)	0–300

### Current practice

#### Provider guidance

Among the pharmacological guidance or recommendations given by PCPs (see **Table 2**), 37.2% reported acetaminophen, followed by nonsteroidal anti-inflammatory drugs (35.5%), tricyclic antidepressants (10.7%), triptans (7.4%) and opioids (6.6%). Only 2.5% of providers reported not providing any pharmacological guidance. Of the non-pharmacological guidance or recommendations (see **Table 2**, n = 63), the majority suggested limited activity (40.8%), followed by lifestyle change (18.4%, e.g., no alcohol, healthy diet, avoid offending activity), non-specific rest (16.5%; this category included a vague endorsement for rest with unclear determination of being bed rest versus limited activity), modified duty status based on patient medical condition (13.6%), and bed rest (9.7%). There were 1.0% who reported not using any non-pharmacological approach. Over 89% of providers utilized both pharmacological and non-pharmacological approaches in treating post-concussive PTH.

**Table 2:** Pharmacological and/or non-pharmacological guidance or recommendations to patient with sustained headache following concussion.

Pharmacological Guidance or Recommendations (N with at least 1 response = 65)		Non-pharmacological Guidance or Recommendations (N with at least 1 response = 63)	
Items	Frequency (%)	Items	Frequency (%)
Acetaminophen (Tylenol)	45 (37.2)	Limited activity	42 (40.8)
NSAIDS	43 (35.5)	Lifestyle	19 (18.4)
Tricyclics/TCA	13 (10.7)	Non-specific rest	17 (16.5)
Triptans	9 (7.4)	Concussion profile	14 (13.6)
Opioids	8 (6.6)	Bed rest	10 (9.7)
None	3 (2.5)	None	1 (1.0)
**TOTAL**	**121 (100.0)**	**TOTAL**	**103 (100.0)**

1: Non-specific rest pertains to responses about rest that were not clearly defined.

2: Multiple responses were allowed.

3: A total of 89.4% of providers reported utilizing both pharmacological and non-pharmacological guidance or recommendations.

With regards to education, there were 70.3% (of 64 clinicians who responded) who acknowledged providing written materials (9 of whom stated ‘sometimes’). Most information was from UptoDate (an evidence-based online resource for clinical decision support for providers), Google, E-profile (an online resource which provides global tracking of SMs with medical conditions, temporary or permanent, that may hinder their readiness to deploy) and the Defense and Veterans Brain Injury Center materials [19].

#### Clinical care

On a Likert scale of 1–10, the PCPs reported a median of 8 (IQR = 6.5, 9; n = 63) for using a similar treatment approach across patients with headache following a concussion. Among the factors providers reported that contributed to differences in care across patients, the most frequent response (33.0%) was symptomatology, followed by injury characteristics (24.2%; e.g., severity of injury, TBI history), patient characteristics (13.2%; e.g., age, patient presentation), comorbidities (12.1%), medications (8.8%), mechanism of injury (7.7%) and treatment failure (1.1%) (see **Table 3**, n = 63). Among providers who reported referring patients to higher level of care (n = 61), the median percentage of patients referred was 30.8% (IQR = 15, 75, range = 0, 100). The factors that guided their referrals included patient symptoms (44.6%), treatment failure (25.0%), injury characteristics (14.1%), neurological deficits (10.9%) and co-morbidities (5.4%) (see **Table 4**). The types of providers to whom patients were referred include neurologists (35.8%) and occupational and physical therapists (both at 3.6%). The types of clinical services to which patients were referred include TBI clinic (39.4%), behavioral health (6.6%), pain management and physical medicine rehabilitation (both at 2.9%), ophthalmology (2.2%), surgery (1.5%) and psychiatry and radiology (both at 0.7%).

**Table 3:** Factors contributing to differences in care from one patient to the next.

Items (N with at least 1 response = 63)	Frequency (%)
Symptomatology	30 (33.0)
Injury characteristics	22 (24.2)
Patient characteristics	12 (13.2)
Comorbidities	11 (12.1)
Medication	8 (8.8)
Mechanism of injury	7 (7.7)
Treatment failure	1 (1.1)
**TOTAL**	**91 (100.0)**

Multiple responses were allowed

**Table 4:** Factors that determine when patient referred to a rehabilitation provider or higher level of care.

Items (N with at least 1 response = 65)	Frequency (%)
Symptomatology	41 (44.6)
Treatment failure	23 (25.0)
Injury characteristic	13 (14.1)
Neurological deficits	10 (10.9)
Co-morbidities	5 (5.4)
**TOTAL**	**92 (100.0)**

**Table 5** outlines clinical follow-up practice. Recommendation for follow-up was reported by all PCPs, with 44.6% recommending 2 or more visits, 15.4% reporting only 1 visit, and 40.0% recommending follow-up depending on the patient’s persistence/worsening of mTBI sequelae. The first recommended follow-up was reported to occur within the first 24 hours by 16.9% of providers, after 1or more week(s) post-injury by 26.2%, between 24–48 hours by 10.8%, between 48–72 hours by 21.5%, in one week by 21.5% and when deemed necessary by 3.1%. The factors that providers reported to determine whether patients are ready to return to duty included symptoms (63.7%, i.e., symptom resolution that may span a gamut of symptom types), activities of daily living (9.9%), time (9.9%), acute concussion profile guidelines (8.8%), and job duties (7.7%, i.e., military occupation specialty) (see **Table 6**).

**Table 5:** Patient follow-up recommendation–count and timing.

Variable	Frequency (%)
**Recommended Follow-up (n = 65)**	
Yes	65 (100.0)
No	0 (0)
**Number of follow-up recommended (n = 65)**	
1 Visit	10 (15.4)
> = 2 Visits	29 (44.6)
Depends on symptoms	26 (40.0)
**When recommend visits to occur (n = 65)**	
< = 24 hours	11 (16.9)
24–48 hours	7 (10.8)
48–72 hours	14 (21.5)
1 week	14 (21.5)
> = 1 week	17 (26.2)
As needed	2 (3.1)

**Table 6:** Factors that determine when patients are ready to return to duty.

Items (N with at least 1 response = 65)	Frequency (%)
Symptoms	58 (63.7)
Activities of daily living	9 (9.9)
Time	9 (9.9)
Profile guidelines	8 (8.8)
Job duties	7 (7.7)
**TOTAL**	**91 (100.0)**

The median time to recovery of concussion patients from symptoms of headache with or without care was 1 month (IQR = 0.5, 3.5, n = 55 and IQR = 0.5, 2, n = 53, respectively). The median percentage of patients reported to follow provider recommendation was 62.5% (IQR = 50, 80, n = 62) (e.g., rest when advised to, take recommended medication). Among patients whom PCPs reported to have followed their recommendation, 83.3% had improvement (n = 54).

#### Comfort providing care

On a Likert scale from 1–10, the median level of comfort PCPs reported in treating PTH was 7.5 (IQR = 6.5, 9; n = 65). The factors reported to influence provider management confidence include level of expertise (41.4%), lack of experience (24.3%), access to specialty care (21.4%) and access to research (8.6%), with 4.3% reporting never being completely comfortable (see **Table 7**, n = 64). Reported challenges related to providing care included patient-related factors (42.7%, e.g., patient compliance, patient expectations, patient fear of stigma), injury-related factors (23.2%, e.g., subjective symptoms, co-morbidities, varying patient presentation), provider-related factors (17.1%, e.g., varying treatment plans, level of expertise, continuity of care) and military-related factors (19.5%, e.g., command, mission requirements) (see **Table 8**, n = 64).

**Table 7:** Factors in determining comfort level of providing care for patients with headache following concussion.

Items (N with at least 1 response = 64)	Frequency (%)
Level of expertise	29 (41.4)
Lack of experience	17 (24.3)
Access to specialty care	15 (21.4)
Access to research	6 (8.6)
Never completely comfortable	3 (4.3)
**TOTAL**	**70 (100.0)**

**Table 8:** Challenges in providing care to patients with post-traumatic headaches post-concussion.

Items (N with at least 1 response = 64)	Frequency (%)
Patient-related factors (e.g., patient follow-up, compliance, expectations)	33 (42.7)
Injury-related factors (e.g., subjective symptoms, co-morbidities, varying patient presentation)	19 (23.2)
Military-related factors (e.g., command, missing requirement, military in general)	16 (19.5)
Provider-related factors (e.g., varying treatment plans, differentiating headache type, level of expertise)	14 (17.1)
**TOTAL**	**82 (100.0)**

## Discussion

Treatment of patients with PTH is complex, given differences in patient presentation, provider training and choice for approach, as well as expectations for recovery. A structured guideline outlining a systematic means of proceeding through recovery, clinical practice and patient outcome assists in the consistency of PTH management among PCPs in a military setting, without undermining the importance of individualized treatment to meet the needs of patients. Our findings provide a preliminary glimpse of the current clinical practice of PCPs treating PTH among SMs.

In this study, a couple of patterns of care described by PCPs align with the DoD recommendations for PTH. This includes recommendation of both pharmacological and non-pharmacological approaches, both of which were given by over 89% of providers, highlighting possibly low bias towards either approach and exemplifying the use of all potential sources as a means to remedy PTH. Future assessment regarding the specific prescribed treatment for a specific type of headache may be needed to further determine the extent of compliance with the recommendations. Special consideration on patient profile, however, will also need to be taken into account, as tailoring treatment to patient needs is critical. In addition to consistency of medical treatment, our findings revealed reports of 100% follow-up recommendation which may give providers the opportunity not only to assess progression of patients with regards to symptomatology and following recommendations, but also to potentially reinforce correct patient behaviors and beliefs regarding PTH recovery through continued patient education. This may, in turn, likely lead to an expedited and relatively successful recovery.

Differences in clinical practice were also captured in the study. Although general medical follow-up care was recommended by all PCPs, the timing of follow-up varied, most of which was more than 1 week. This may reflect the general practice of the study providers with recommending patient follow-up, which may be case-specific. As expedited and successful recovery often depends on timely treatment as well as regular monitoring of patient response to such treatment, embedding the importance of follow-up and when to follow-up in clinical training to treat PTH may help in the recovery progression. Although the care given by each PCP across their patients was generally similar for most, the data did not have the granularity needed to determine the appropriateness of these approaches. For PCPs whose approach differed across patients, the factors that determine these differences in care also varied. This may perhaps be due to personalized care in which patient profile, as well as specific injury, might have necessitated more individualized treatment (e.g., failure of response to primary treatment). Providers might be operating within the parameters of the clinical practice guideline they use; however, it is difficult to know this with certainty without a more granular approach to identify the effectiveness of various clinical provider guidelines. The DoD CR was created to ensure standard treatments for the Military Health System. With regards to referrals to higher levels of care, the factors that determine this consideration also varied, highlighting a need for a more systematic and objective means to determine referrals to higher levels of care or rehabilitation such as that provided in a standardized clinical guidance.

Most study providers were comfortable in treating headaches of concussed SMs, and only two indicated not being completely comfortable. Nonetheless, almost all reported challenges in providing care, majority of which are modifiable, such as patient-related (e.g., “patient compliance”, “patient expectations”) and provider-related (e.g., “varying treatment plans,” “level of expertise”) factors that can be directly addressed through education. As the challenges in providing care contribute to the level of comfort in treating patients, ameliorating some of the challenges that may pose as obstacles to PCPs may subsequently ease comfort in patient treatment. This further supports the utility of concussion education, in this case, to address the challenges that may hinder confidence among PCPs treating PTH patients.

### Limitations

There were limitations to the present study that need to be considered. First, certain details pertinent to PTH treatment were not asked of providers. For example, the semi-structured interviews did not inquire whether clinical recommendations were based on headache type (i.e., migraine, tension-type, cervicogenic, neuropathic) to further gauge compliance with the DoD CR. However, this less specific inquiry allowed for more provider-led responses. Additionally, when study providers offered the term “rest” as a response, no further inquiries were made to determine how individual providers defined “rest.” This might have hidden some inconsistencies in treatment with regards to the type and duration of “rest”. This study was also designed to survey PCPs’ treatment practices of PTH and did not account for factors that might influence or modify treatment. For instance, the semi-structured interview did not directly ask providers whether they altered their clinical recommendations according to patient history (e.g., headache trigger, co-morbidities, individual or family history of headache, medications). Although such a follow-up question might provide a better depiction of changes in treatment over time, the authors felt this line of inquiry, though important, was outside the scope of the present study. Future studies that provide a more granular assessment of clinical practice for the management of PTH may address these matters and provide a more accurate depiction of current clinical practices. Further investigation that evaluate patient compliance with suggested provider recommendations and follow-up may also be indicative of the level of effectiveness of PTH management whether in or out of compliance with the DoD CR. Reported or perceived practice may also not be congruent to actual practice; however, this is inherent of most studies that assess self-reported data and is a limitation of broader research using such data. Further, this study evaluated PCPs treating active duty SMs and, thus, results are generalizable only to such, or similar, populations. Nonetheless, the study contributes to the limited research on this topic in this group. We also presented our qualitative data grouped subjectively into common themes and quantified accordingly; however, such categorization was based on a compilation of input from a team with expertise in medicine, epidemiology and mTBI/concussions and PTH to minimize miscategorization. Last, based on the limited sample size, we also could not evaluate our results based on potentially important modifiers such as type of provider (e.g., civilian vs. military or physicians vs. nurse practitioners), years of practice, or initially prescribed pharmacological treatment as it contributes to follow-up time (e.g., the time for tricyclic antidepressants to be effective versus nonsteroidal anti-inflammatory drugs/analgesic is unlikely to be seen within 72 hours and may influence follow-up time). Nonetheless, our findings provide value as it emphasizes the importance of training and education of providers in the treatment of PTH, as well as the potential to revise and improve the clinical recommendation to enhance training/education, and, ultimately, patient outcome.

## Conclusions

The findings of this study present an account of the current clinical practice of PCPs in the management of PTH, and support the use of a formal, structured CR for the management of PTH among SMs to guide PCPs in a military setting. Having a standardized approach across military treatment facilities may assist in the consistency of medical care given to patients with PTH, particularly as providers in the military setting often transition to different assignments. Such guidance should be designed to educate both provider and patient in their expectations regarding the management of PTH among SMs, as well as other aspects of the provider experience (e.g., diversity of experience as a PCP in the military health system). Proceeding research will specifically examine the potential benefits of training on the DoD CR for PTH among SMs.

## Supporting information

S1 Table Provider interview questions by time of interview. (DOCX)

## Abbreviations

PTH: Post-traumatic headache
mTBI: Mild traumatic brain injury
SMs: Service members
PCPs: Primary care providers
DoD: Department of Defense
CR: Clinical recommendation
SD: Standard deviation
IQR: Interquartile range
