# Supplementary material for: Current patterns of primary care provider practices for the treatment of post-traumatic headache in active duty military settings
Source: PLoS One. 2020 Jul 24;15(7):e0236762. doi: 10.1371/journal.pone.0236762 (PMC7380628; doi:10.1371/journal.pone.0236762)
Supplement: S1 Table — (DOCX) [file pone.0236762.s001.docx]

**S1 Table. Provider interview questions by time of interview**

| **BACKGROUND** |
| --- |
| *As a healthcare provider, what is your professional role? (e.g., nurse, nurse practitioner, physician, physician assistant) |
| *For how many years have you been practicing? |
| *For how many years have you been working as a provider in the Military Health System? |
| *In which facilities and departments have you worked where you have seen patients with headache following concussion? |
| *For how many years have you been working in this particular department? |
| *For how many years have you been treating patients with headache following concussion? |
| **CURRENT PRACTICE** |
| *On average, how many patients with headache do you treat per week? (recalculated as per month) |
| *On average, how many patients with concussion do you treat per week? (recalculated as per month) |
| *About how many patients with acute concussion have you treated in your clinical practice ever/overall? |
| *What pharmacological and/or non-pharmacological guidance or recommendations do you provide to a patient who has sustained a headache following concussion? |
| *On a scale of 1 to 10, how similar is the approach you provide across patients with headache following concussion? (1 is never the same and 10 is exactly the same)?  *What factors contribute to differences in care you provide from one patient to the next? (e.g., comorbid diagnoses, available time with patient, patient interest) |
| *Do you typically recommend any medical follow-up to patients?  *If yes:  *How many follow-up visits with the patient do you recommend?  *When do you recommend the visits occur (e.g., one day later)?  *Other comments about follow-ups. |
| *What percentage of your concussion patients with headache do you refer to a rehabilitation provider and/or higher level of care?  *What factors determine when you refer a patient to a rehabilitation provider or higher level of care?  *To what type of provider would you typically refer a concussion patient with headache? |
| *What factors determine when you consider a patient ready to return to duty? |
| *Do you provide any written materials to patients as part of your guidance?  *If yes, what materials do you provide? |
| *On a scale of 1 to 10, how comfortable are you providing care for patients with headache following concussion (1 is not at all comfortable and 10 is completely comfortable)? Please explain your response. |
| *Can you describe some of the challenges in providing care for patients with headache following concussion? Please explain your response. |
| **PATIENT CHANGE AND COMPLIANCE WITH TREATMENT** |
| *In your view, how long (e.g., how many weeks, months) does it take for concussion patients to typically recovery from symptoms of headache *regardless of medical care*? |
| *In your view, how long (e.g., how many weeks, months) does it take for concussion patients to recovery from symptoms of headache as *a result of the care you provide*? |
| *What aspects of your care do you think are most influential in supporting positive patient outcomes? |
| *In your view, what percentage of your patients follow recommendations you provide (e.g., does the individual rest when advised to, take recommended medication)? |
| *In your view, did patients who followed your recommendations show better, worse or the same recovery compared to recovery of patients who did not follow the recommendation? Please explain. |
